# Genes Encoding Callose Synthase and Phytochrome A Are Adjacent to a *MAP3K*α**-Like Gene in *Beta vulgaris* US H20

**DOI:** 10.1155/2011/370548

**Published:** 2011-06-07

**Authors:** L. David Kuykendall, Jonathan Y. Shao

**Affiliations:** Molecular Plant Pathology Laboratory, Agricultural Research Service, US Department of Agriculture, Plant Sciences Institute, Beltsville Agricultural Research Center, 10300 Baltimore Avenue, Building 004, Room 120, BARC-West, Beltsville, MD 20705, USA

## Abstract

*MAP3Kα*, a gene that encodes a key conserved protein kinase, is responsible for initiating a rapid cascade of cellular events leading to localized cell death. Hypersensitive response, as it is termed, enables genetically resistant plants to limit microbial invasion under the right environmental conditions. Since knowledge of close physically linked genes is important for genome analysis and possibly for improving disease resistance, systematic DNA sequence analysis, gene annotation, and protein BLASTs were performed to identify and characterize genes in close physical proximity to a *MAP3Kα*-like gene in *Beta vulgaris* L. US H20. On the same 125 Kb BAC, callose synthase (*BvCS*) and phytochrome A (*PhyA*) genes were within 50 Kb of *MAP3Kα*. The close physical linkage of these genes may result from selection for coordinated responses to disease pressure. Bert, a new chromodomain-carrying *gypsy*-like LTR retrotransposon, resides within an intron of the *BvCS* gene, where it is transcribed from the opposing strand.

## 1. Introduction

A plant gene, *MAP3K*α**, produces a highly conserved protein product that activates hypersensitive response, a mechanism underlying R gene-mediated disease resistance [1]. In tobacco and in tomato, MAP3K*α* activates cascades of enzymatic activations leading to a crescendo that is apoptosis or programmed cell death, a critical component of R gene-mediated disease resistance [1].

Research done on the crop plants, tomato and tobacco, as well as that performed on the model plant system *Arabidopsis thaliana* L. Heynth, over a 20-year period in a several laboratories, has presented adequate evidence that a particular gene called *MAP3K*α** is centrally important to R gene-mediated plant disease resistance [1]. In essence, a pathogen elicitor causes a conformational change in a plant protein initiating a cascade reaction leading to the so-called hypersensitive response, a primary countermeasure deployed by plants in order to effectively resist pathogen invasion. This key process is controlled by the protein product of *MAP3K*α*.*

In the genome of *Arabidopsis thaliana*, large-scale duplication of genetic regions followed by selective gene loss has created a recognized network of chromosomal synteny [2]. By developing physical genetic maps based on ESTs, Dominguez et al. [3] discovered conserved synteny with *Arabidopsis* among genomes of four phylogenetically divergent eudicot crops, namely, sugarbeet, potato, sunflower, and plum. 

In our previous study, complete BAC sequence analysis identified two core plant genes, *CaMP* and *CKI*, tightly physically linked to the disease resistance controlling gene *NPR1* and established a conservation of microsynteny between the* NPR1* gene regions of sugarbeet and other eudicots [4]. Also, an *HSF *gene adjacent to, just 2 Kb downstream from, *NPR1* in sugarbeet and whose close microsynteny is conserved in four out of five eudicots examined, encodes a DNA-binding HSF protein similar to that specified by gene *HSFA9* that controls early leaf morphogenesis in sunflower [4, 5]. 

The central role of *MAP3K*α** in positively and globally activating hypersensitivity to pathogen invasion, as an effective defense mechanism in response to an elicitor(s) produced by the pathogen, suggests the possibility of enhancing disease resistance in plants by genetic manipulation of expression of the* MAP3K*α** gene. As a step toward identifying genes localized near the* MAP3K*α** gene in sugarbeet, a bacterial artificial chromosome (BAC) library was screened using PCR and gene-specific primers, and a clone, SB3, was identified as carrying a *MAP3K*α**-like gene. An expressed sequence tag, EST clone BQ585699, was instrumental in designing primers used for discovery of the *MAP3K*α**-like gene from sugarbeet US H20. The *Beta vulgaris MAP3K*α**-like gene encodes a predicted protein product with high similarity to protein products encoded by disease resistance-orchestrating mitogen-activated protein kinase kinase kinase genes in tomato, tobacco and the model plant species *Arabidopsis thaliana* (in preparation). 

We report herein new information regarding the gene content and organization of a 125 Kb contiguous fragment of sugar beet genomic DNA contained in a sugarbeet BAC carrying* MAP3K*α**. Our discovery of close physical linkage of *MAP3K*α** with genes encoding callose synthase,* CS*, and phytochrome A,* phyA*, is described for the first time. Discovery of a novel chromodomain-carrying *gypsy*-like LTR retrotransposon, Bert, is also described.

## 2. Materials and Methods

### 2.1. DNA Sequencing

Genomic DNA of *B. vulgaris* hybrid US H20 [6] (PI 631354), with an estimated 750 Mb genome size, had previously been used to construct a BAC library [7]. About 34,500 clones comprised the BAC DNA library, average insert size was about 120 Kb, providing about 6.1X genome coverage [7]. Primers designed based on the DNA sequence of GenBank accession, BQ585699, an EST sequence encoding for a *B. vulgaris* MAP3K, were utilized to screen and identify a *BvMAP3K*α**-carrying BAC (manuscript in preparation). The presence of a complete genomic MAP3K*α* gene was established by DNA sequence analysis of BAC clone SB3.

BAC sequencing was completed at Washington University's Genome Sequencing Center in St. Louis, Missouri, USA (http://genome.wustl.edu/). The BAC clone SB3 was provided to the Genome Sequencing Center as a glycerol stock. Purification, library construction, shotgun cloning, and sequence analysis were performed on a sufficient number of random subclones to provide about 9.5X coverage. ABI 3730 capillary sequencers were used. Data was assembled using the phred/phrap suite (http://www.phrap.org/).

### 2.2. Gene Annotation

Analysis of sequence data was performed using Lasergene (DNASTAR, Madison, WI) for assembly, and NCBI BLAST [8]. The 125 Kb sequence was screened for coding sequence using a combination of the following programs: GeneMark [9, 10] for eukaryotes (http://exon.gatech.edu/GeneMark/eukhmm.cgi), Augustus (http://augustus.gobics.de/), and FgenesH (http://softberry.com/). *Arabidopsis thaliana*, *Solanum lycopersicum*, and *Medicago truncatula* were chosen as models where possible and default settings were used for each gene finder. BlastP searches were performed at the National Center for Biotechnology Information (NCBI) web site (http://www.ncbi.nlm.nih.gov/BLAST/). Manual curation of proteins was performed using Lasergene MegAlign and EditSeq sequence analysis software. Where applicable Simple Modular Architecture Research Tool (SMART) [11] database (http://smart.emblheidelberg.de/) was used to identify protein domains and motifs. ARTEMIS (http://www.sanger.ac.uk/Software/Artemis) was used to collate data and facilitate annotation. LTR retrotransposon analysis was done using LTR STRUC program [12]. 

### 2.3. Comparative Similarity Analysis

BlastP searches of predicted protein products of sugar beet genes were performed at http://www.ncbi.nlm.nih.gov/BLAST/, similarity analysis of proteins was performed using the Mega program (http://www.megasoftware.net/) using neighbor joining method and ClustalX alignment program.

## 3. Results

**Figure 1:**
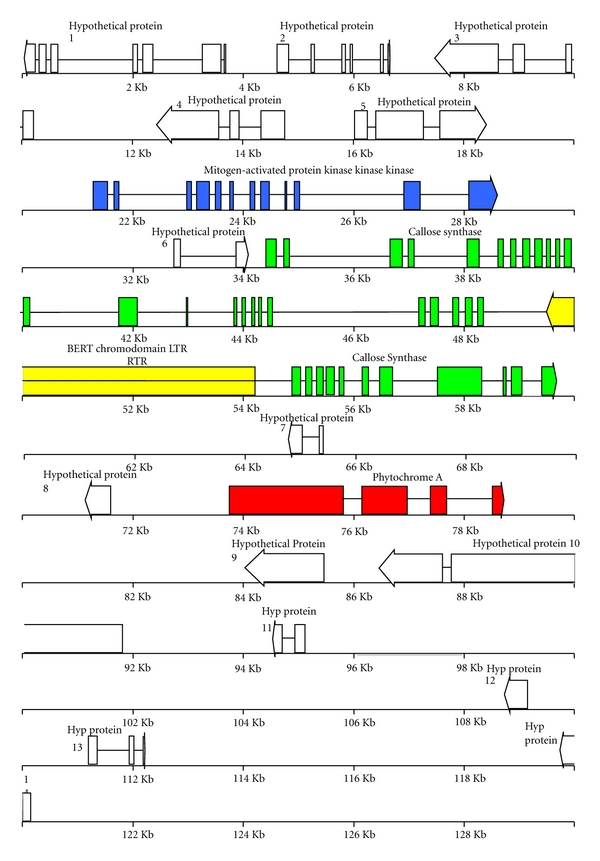
Schematic representation of features annotated on the 125 Kb genomic *MAP3K*α**-like gene carrying-BAC SB3 from sugarbeet (Genbank accession number GU057342). [Blue, *MAP3K*α** core plant genes involved with hypersensitive response due to genetic resistance: Green, *BvCS* callose sythase; Yellow, Bert chromodomain *gypsy*-like retrotransposon with Grey LTRs and Red, *PhyA *phytochrome A gene, and [Empty white] unidentified ORFs which encode only hypothetical or unknown proteins]. The predicted genes begin with a bar and ends with an arrowhead, thus indicating the direction of transcription.

A 125 Kb contiguous fragment of sugarbeet chromosomal DNA contained in SB3, a sugarbeet BAC carrying a *B. vulgaris MAP3K*α**-like gene, was sequenced and fully annotated (GenBank accession GU057342 is scheduled for release on 10/05/10). Bioinformatics tools Fgenesh, GeneMark, and Augustus were used as gene finders. Designated gene names and predicted functions of deduced amino acid sequences, where possible, are presented in Table 1 and a visual representation of exon structure is depicted in Figure 1. Within the 125 Kb contiguous fragment of sugarbeet genomic DNA, eighteen open reading frames (ORFs), or protein-encoding regions, were identified. Only three ORFs were predicted to produce protein products with high amino acid sequence similarity to known products of core plant genes (Table 1). In addition to the three core plant genes, the 125 Kb contiguous fragment of genomic DNA was found to carry an insertion of a novel chromodomain-carrying *gypsy*-like LTR retrotransposon, which we call “Bert” in keeping with widely accepted nomenclature of similar transposable elements in other plants. Bert's predicted polyprotein has a C-terminal chromodomain, and Bert, localized within an intron of a 36-exon callose synthase gene, is transcribed from the opposing strand. The other fourteen putative genes were predicted to produce proteins that either lack a known function or are ancient or defective retrotransposons. 

**Figure 2:**
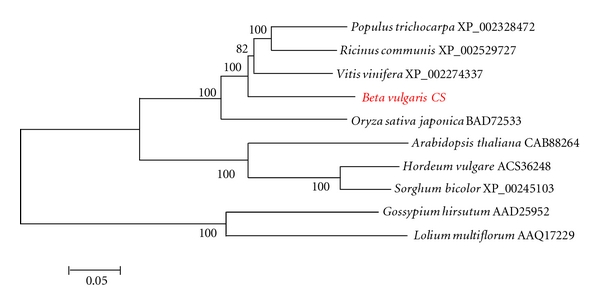
Neighbor-joining similarity tree based on MegAlign amino acid sequence alignments (not shown) of predicted products of callose synthase genes. These proteins had some of the best BlastP matches with the protein product of the *BvCS *gene.

In addition to *MAP3K*α**, another core plant gene within the 125 Kb contiguous fragment of sugarbeet genomic DNA carried by BAC clone SB3 was a 36-exon callose synthase gene, *BvCS*, that encodes a *β*-1,3-glucan synthase protein having a conserved glucan synthase domain (*E* = 8.7*e* ^−148^) from amino acid positions 1159–1779 and various transmembrane domains by SMART. The predicted BvCS protein has high amino acid sequence alignment similarity (Table 1) with the protein product of *CS5*, a male fertility-controlling gene of *Arabidopsis thaliana* [13] whose product is also involved in callose deposition in response to wounding [14]. The predicted protein product of *BvCS* also is very similar in amino acid sequence alignment to CS5-like gene products in castor bean, grape, and poplar (Figure 2).

The *BvCS* gene is localized between the *MAP3K*α** gene and a phytochrome A gene, *PhyA*, encoding a photoreceptor and transcriptional activator which migrates to the nucleus when activated by light of the appropriate wavelength. The *MAP3K*α**, *BvCS1*, and *PhyA* genes are all/ each transcribed from the positive strand (Figure 2), but *Bert*, transcribed from the negative strand, is localized within one of the 31 introns of *BvCS*. LTR_STRUC analysis revealed that, at about 434 bp in length, *Bert*'s LTRs are 100% identical in nucleotide sequence, and *Bert*'s single ORF encodes a 1,558 amino-acid protein.

Beginning at about 45 Kb downstream of the *MAP3K*α** gene but only about 14 Kb downstream of gene *BvCS*, another core plant gene, *PhyA*, encodes, in four exons, a phytochrome A-like protein with a PHYTOCHROME domain from amino acid positions 413 to 592, as well as other protein domains. SMART also confidently predicted the following: (1) a GAF domain, characteristic of phytochromes and cGMP-specific phosphodiesterases, from amino acids 219 to 412, (2) a PAS-2 domain characteristic of proteins with roles in sensory perception, protein-chromo-phore linkage, and regulation of transcription from amino acids 70 to 186, (3) two PAS domains from amino acids 620 to 686 and from amino acids 750 to 819, (4) a histidine kinase domain from amino acids 895 to 959, and (5) an ATPase domain, characteristic of histidine kinases, DNA gyrase B, and phytochromes, from amino acids 1007 to 1119.

**Figure 3:**
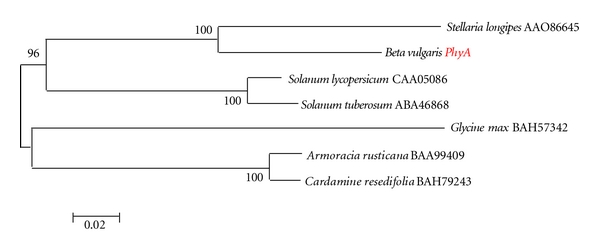
Neighbor joining similarity tree based on MegAlign amino acid sequence alignment (not shown) of predicted products of phytochrome A genes. These proteins had some of the best BlastP matches with the protein product of the *BvPhyA *gene.

The predicted protein product of the *phyA* gene gave numerous BLAST hits *E* = 0.0, indicative of a good match by amino acid sequence alignment, to phytochrome A proteins. Relative to the product of sugarbeet *phyA*, the phytochrome A protein with the most similar amino acid sequence alignment was from *Stellaria longipes* or longstalk starwort (Table 1, Figure 3). In a different subclade, were phytochrome A proteins from *Solanum lycopersicum*, tomato, and *Solanum tuberosum*, potato, based on an amino acid sequence alignment similarity tree (Figure 3), obtained using complete amino acid sequence alignments and MegAlign (not shown). A distinct clade of phytochrome A proteins contained proteins from *Armoracia rusticana*, horseradish, and *Cardamine resedifolia*, an alpine wildflower.

Domain architecture of the predicted protein products BvCS and BvPhyA is illustrated in Figure 5. Ending at about 50 Kb downstream of *MAP3K*α**, the sugarbeet *phyA* gene, is interrupted by three introns (Figure 1). Close physical proximity of genes *MAP3K*α**, *BvCS*, and *PhyA* was discovered in *B. vulgaris*. *MAP3K*α** encodes an alpha-like mitogen-activated protein kinase kinase kinase of the type that orchestrates the hypersensitive response responsible for genetic disease resistance. Our in silico analyses of the predicted products of the two nearby core plant genes show unequivocably that (1) *BvCS1* encodes a callose synthase of the type responsible for normal pollen tube function and for response to wounding and that (2) *PhyA* encodes a Phytochrome A-like light signal receiver which has both a histidine kinase domain and an ATPase domain not too dissimilar to that found in the agriculturally important *Solanum* genus containing potato and tomato.

**Figure 4:**
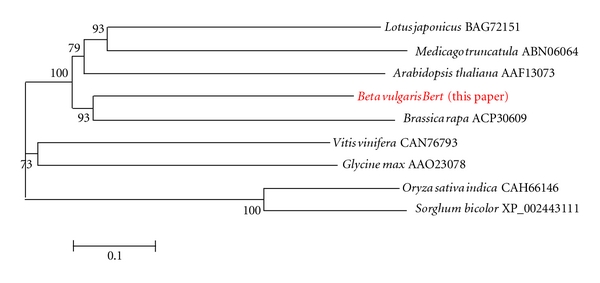
Neighbor-joining similarity tree based on MegAlign amino acid sequence alignments (not shown) of predicted products of chromodomain-containing *gypsy*-like LTR retrotransposon genes.

**Figure 5:**
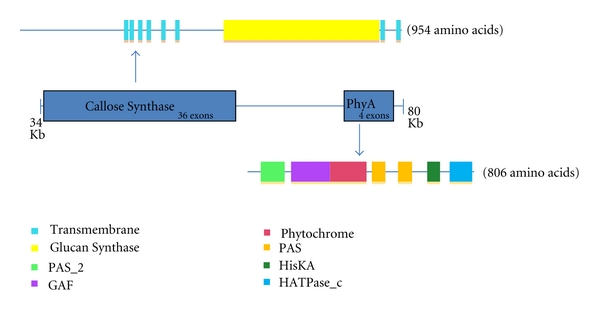
An illustration of the domain architecture of predicted protein products of the *BvCS* and *PhyA* genes downstream from the *MAP3K*α** gene.

*Bert*, 5.5 Kb in length, is a novel chromodomain-carrying, *gypsy*-like LTR retrotransposon in a single exon [as expected] (Table 1, Figure 1). Nucleic acid Blast at NCBI produced an E = 0.0 alignment with a soybean retroelement polyprotein AAO23078. A similarity tree (Figure 4) shows that *Bert* also aligns best with a retroelement polyprotein in *Brassica rapa* based on complete amino acid sequence alignments obtained using MegaAlign (not shown). Genome analyses of model plant species within *Arabidopsis*, *Lotus*, and *Medicago* has produced evidence for other predicted retroelement poly-proteins similar to *Bert* in terms of a similarity analysis of complete amino acid sequence alignments (Figure 4). Compared with the above, *Bert* is less similar to a complete *gypsy*-like LTR retrotransposon from sugarbeet we previously described, *Schmidt* [15] in overall amino acid sequence alignment (not shown). Nucleic acid Blast alignment with other retroelements at the Plant Repeat Database (PRD) produced a match with an *E* value equal to 2.2*e* ^−77^ with “rn_460_239” from Graminaceae, the grasses.

## 4. Discussion

In this study, analysis of genes that are very physically close to the *MAP3K*α** gene of *B. vulgaris* revealed, for the first time, that a callose synthase gene, whose product likely plays major structural, defense, and developmental roles, and a *PhyA *gene, encoding a phytochrome A protein kinase with tripartite roles in light perception, signal transduction, and nuclearly-localized activation of multigene transcription in response to light availability [16, 17], are adjacent to the *MAP3K*α** gene, whose protein product orchestrates the hypersensitive response, the primary plant genetic resistance countermeasure. Glucan synthase (GS), or uridine-diphosphate glucose: (1–>3)-*β*-D-glucan 3-*β*-D-glucosyl transferase, interacts with phragmoplastin, UDP- glucose transferase, a Rho1-like protein and possibly annexins, depositing callose in different locations in response to specific abiotic, biotic, and developmental signals [13].

The BERT retrotransposon transcribed from the negative strand, within an intron of *BvCS*, is probably active since its long terminal repeats are 100% identical. Its transposition, probably stress-induced, would likely occur under conditions of severe stress, such as tissue culture, potentially resulting in random mutagenesis, or “somaclonal variation,” as the earlier literature described the phenomenon.

Within a diverse family, *CS* genes, located on different chromosomes, encode large transmembrane proteins in ORFs interrupted by 1 to 49 introns [13]. Callose, a 1, 3-*β*-D-glucan with a few 6 linked branches composed of glucose monosaccharide linked by 1, 3 beta linkages, is formed in the cell wall and many other places depending on the stage of development. Callose is usually found in the immediate vicinity of the cell wall where it serves as a plugging mechanism whenever the cell wall suffers disruptive stress such as herbivory by insects or other wounding [18].

 Callose is deposited between the plasma membrane and the cell wall after exposure to either abiotic or biotic stresses. As a programmed plant cellular response, callose deposition is usually an effective means of resisting microbial attack, insect feeding, or physical stress. By very rapidly synthesizing and depositing callose as plugs, drops, or plates in close proximity to an invading pathogen or a damaged area, the plant cell prevents more serious damage. Callose deposits, often referred to as papillae, may contain minor amounts of other polysaccharides, phenolic compounds, reactive oxygen inter-mediates, and proteins [14]. Papillae are believed to literally wall off microbial intruders [14].

Prerequisite to and then concurrent with callose deposition, a rapid influx of Ca^2+^  into the cytoplasm occurs. Ca^2+^  acts as a second messenger that transmits signals received from receptors on the cell surface, including elicitors from pathogens, to target molecules in the cytosol. Ca^2+^ helps to initiate the well-documented oxidative burst and activates cascades and other defense responses culminating in the hypersensitive response (HR), programmed cell death, or apoptosis—the ultimate cellular defense [19]. 

During cytokinesis, callose deposition to the developing cell plate may be important for septum formation [18]. It has also been hypothesized that callose controls cell-to-cell movement of molecules through the plasmodesmata. Callose is known to plays key roles in pollen grain formation and pollen tube growth [13]. 

The CS protein product encoded by the *CS* gene adjacent to MAP3K*α* in BAC3 is most similar in amino acid sequence alignment to the product of *CS5*, callose synthase 5 (Table 1). The amino acid sequence alignment of the *Arabidopsis CS5* gene product may be an atypical outlier from the group as a whole (not shown) but, consistent with the prediction, there is an observed high degree of amino acid sequence similarity between the predicted protein product of *BvCS* and gene products of a number of *CS5*-like genes of various plant species (Figure 2). There is a close physical linkage of the *MAP3K*α** gene with a *BvCS* gene whose product is herein predicted as a callose synthase 5. Largely expressed only in male germ cells, the *CS5* gene encodes a so-called “male-specific” beta 1, 3 glucan synthase and is needed to produce the temporary callose walls that separate the developing microspores. The callose that is deposited on the surface of microsporocytes serves as a temporary cell wall [13]. 

Phytochromes are photoreceptors that regulate plant photomorphogenesis, growth, or development that is stimulated by red, infra red, and blue light. Photoreceptors monitor intensity, direction, quality, and duration of light [16]. Phytochromes absorb at 600–800 nm and optimize the capture of light energy needed for photosynthesis and other core metabolic processes. Phytochromes function during all of the stages of the cell and organismal life cycles and their primary roles are to acquire information on the light environment of a plant and to provide the plant with the means to adapt to change, both expected and unexpected, in the supply of light energy [17].

Previously a hypothesis was proposed [4] that conserved microsynteny of certain core plant genes in eudicots may correlate with either their subcellular localization or with related function as is often the case with clusters of genes in bacteria. In this present study, the cellular roles ascribed to the predicted protein products of the three core plant genes found clustered on sugarbeet genomic DNA carried by SB3 correlate well with a clear need for coordinated expression. *MAP3K*α** initiates the hypersensitive response, apoptosis, leading to effective genetic disease resistance. Pathogen elicitor-activated MAP3K*α* functions to phosphorylate other protein kinases that, in turn, phosphorylate other protein kinases and so on [hence the “3K” or kinase kinase kinase terminology]. Signal transduction cascades occur concurrently very rapidly in response to the detection of a recognized specific pathogen. If the plant has the particular resistance gene that encodes a product that directly or indirectly responds to pathogen elicitor(s), the resulting hypersensitive response leads to effective disease resistance by a mechanism(s) that are still not yet completely clear. 

*CS* expression, controlled in the plant nucleus, is essential for male gametophyte viability/fertility [13, 20]. It should be noted that in *Arabidopsis* there are twelve *CS* genes expressed in different plant tissues and whose products have diverse roles [21]. *Oryza sativa* subspecies *japonica* has a type of *CS* gene, exemplified by 55771366 responsible for a protein product that has high amino acid similarity to products predicted for *BvCS * and other *CS5*-like genes in *Populus tricarpa*, *Ricinus communis*, and *Vitis vinifera* (Figure 2). All are part of the plant's response to biotic as well as to abiotic stresses. Since some defense responses exhibit a well-documented requirement for phytochrome activation [22–25], coordination of systemic defense responses with energy availability is irrefutable and, consequently, both the *PhyA* gene and the *BvCS* gene localized near *MAP3K*α** in sugarbeet likely play important roles in stress responsiveness.

Including *Bert*, a total of 15 retrotransposon- (RT)-like or hypothetical genes lie within the approximately 125 Kb BAC carrying sugarbeet genomic DNA specifying a small core gene cluster consisting of *BvMAP3K*α**, *BvCS*, and *PhyA* genes, all in an about 60 Kb long genomic DNA region from *Beta vulgaris*. Thus the immediate region around the small core gene cluster is rich in repetitive elements since several insertions of mobile genetic elements have occurred during both horizontal gene acquisition and vertical evolutionary descent. ORFs originating from either retrotransposons or viruses, from DNA transposons and other repetitive elements, need not be considered disruptive of colinearity of core genes nevertheless. This about 125 Kb contiguous genomic DNA fragment, rich in highly-degraded repetitive elements, contains only a single one-exon ORF, *Bert*, likely encoding an active* gypsy*-like CHR domain LTR retrotransposon polyprotein. *Bert* has some similarity to the previously described gypsy-like retrotransposons *Schmidt* [15] and *Beetle1*, a new chromodomain LTR retrotransposon* of Beta procumbens* [26], but these two previously described LTR retro-transposons are more similar in predicted amino acid sequence alignment with each other than they are with *Bert*, consistent with *Bert*'s novelty. Nevertheless, the insertion of *Bert *into an intron of the *BvCS* gene does not alter the conclusion that there are three intact core essential genes in close physical proximity, transcribed in the same direction and with protein products predicted to play either direct or indirect roles in activation of defense response mechanisms. These findings are consistent with our hypothesis concerning the “raison d'être” for gene clustering.

By comparing the orthologous *NPR1*-carrying regions of *Medicago truncatula* and *Populus trichocarpa* with that of *B*. *vulgaris*, we discovered conserved microsynteny for *NPR1*, *CaMP*, and *CK1PK *genes [4]. Conserved microsynteny of *NPR1*, *CaMP*, and* CK1PK* in *B. vulgaris*, *M. truncatula*, and* P. trichocarpa *may help coordinate expression [4]. More recently, very close physical linkage in monocots of *Bx1* and *Bx2*, and if *Bx3* and *Bx4* genes-encoding enzymes, responsible for steps in benzoxazinoid synthesis, suggests functional clusters related to coordinated expression for this biosynthetic pathway [27].

Close physical proximity of key core plant genes suggests there has been a positive selection for the arrangement. Close physical linkage of the three core plant genes, *BvMAP3K*α**, *BvCS*, and *PhyA*, even if observed only in sugarbeet, may hypothetically facilitate coordinated expression of genes critical to plant defense response with other cellular and organismal processes including adaptation to abiotic as well as biotic stress, efficient as well as timely response to change in light availability, reproduction, and even programmed cell death as necessary to protect the plant from the spread of an erstwhile destructive avirulent pathogen.

Reverse transcriptase (RT) PCR, quantitative qPCR expression studies, or global transcriptional profiling will furnish information relevant to the question of the interrelatedness of plant defense, cellular integrity, and reproductive fitness, as well as light responsiveness roles of MAP3K*α*, callose synthase, or phytochrome A. Upregulated expression plant of genes in response to pathogens and/or oxidative stress requires intense investigation.

In summary, in addition to *MAP3K*α** and the closely physically linked *BvCS* and *BvPhyA* genes herein described for the first time, the 125 Kb* MAP3K*α**-carrying *Beta vulgaris* BAC SB3 also encodes *Bert*, a new chromodomain gypsy-like retrotransposon, and has 14 other as yet undefined features. Whereas some of these ORFs produce predicted proteins with probable retrotransposon origins deduced from BLAST analysis, the other unidentified ORFs had predicted protein products without any known function. 

Close physical proximity of *MAP3K*α**, *BvCS1*, and *PhyA* suggests positive selection in the history of breeding and genetic hybridization of *Beta vulgaris* for coordinated expression of these particular genes whose presumably essential products either control genetic plant disease resistance, callose synthesis or responses to light.

## Figures and Tables

**Table 1:** Genes encoded within BAC SB3, amino acid alignments, and predicted function of predicted protein products and designations.

Gene	Protein product molecular weight (KDal)	Best BLAST amino acid sequence hit^a^	*E* value	Similarity	Designation^b^
Hp1	—	—	—	—	hypothetical^c^
Hp2	—	—	—	—	hypothetical^c^
Hp3	—	—	—	—	hypothetical^c^
Hp4	—	—	—	—	hypothetical^c^
Hp5	—	—	—	—	hypothetical^c^
MAP3K*α*	73.9	AAS78640	0.0	452/692	MAP3K*α*
Hp6	—	—	—	—	hypothetical^c^
Callose synthase	220.6	AAK49452	0.0	1661/1920	CS5-like
Gypsy-like LTR RTR4, with chromodomain	179.6	AAF13073	0.0	965/1471	BERT
Hp7	—	—	—	—	hypothetical^c^
Hp8	—	—	—	—	hypothetical^c^
Phytochrome A	124.3	AAO86645	0.0	1034/1121	PhyA
Hp9					hypothetical^c^
Hp10					
Hp11					
Hp12					
Hp13 Hp14					

^a^GenBank accession number or protein ID of the best BLAST hit, followed by the *e* value and percent (similar/total amino acids) similarity between the query and the best hit.

^b^Designation based on a deduction possible by use of bioinformatics tools listed in Section 2. Functional classification based on the result of protein BLAST search.

^c^N.A.: not applicable; putative function of the product not identified.
